# *Phoenix dactylifera* Mucilage and Polyvinyl Alcohol-Based Plaster Gel for Nicotine Delivery

**DOI:** 10.1155/adpp/1305224

**Published:** 2025-05-21

**Authors:** Thipapun Plyduang, Pattwat Maneewattanapinyo, Chaowalit Monton, Wiwat Pichayakorn, Kamon Panrat, Jirapornchai Suksaeree

**Affiliations:** ^1^School of Pharmacy, Walailak University, Nakhon Si Thammarat 80160, Thailand; ^2^Drug and Cosmetics Excellence Center Walailak University, Nakhon Si Thammarat 80160, Thailand; ^3^Department of Pharmaceutical Chemistry, College of Pharmacy, Rangsit University, Muang, Pathum Thani 12000, Thailand; ^4^Drug and Herbal Product Research and Development Center, College of Pharmacy, Rangsit University, Muang, Pathum Thani 12000, Thailand; ^5^Department of Pharmacognosy, College of Pharmacy, Rangsit University, Muang, Pathum Thani 12000, Thailand; ^6^Department of Pharmaceutical Technology, Faculty of Pharmaceutical Sciences, Prince of Songkla University, Hat-Yai, Songkhla 90112, Thailand; ^7^Pharmaceutical Laboratory Service Center, Faculty of Pharmaceutical Sciences, Prince of Songkla University, Hat-Yai, Songkhla 90112, Thailand

**Keywords:** date palm, mucilage, nicotine delivery, plaster gel

## Abstract

The potential uses of extracting mucilage from plant sources have led to much research in this field. One possible source of mucilage for agri-food-pharma utilization is the fruits of the *Phoenix dactylifera*, date palm. For developing the plaster gel loaded with nicotine, we, therefore, applied the mucilage from date palm fruits as a gel-forming agent. Other components, however, might be added to increase its properties. Response surface methodology was used for quantifying the effects of a range of variables (date palm mucilage, PVA, and glycerin) on physicochemical parameters (pH value, viscosity, drying time, ultimate tensile strength, elongation at break, and drug content). The optimal formulation was 3.5%:1.8%:30% w/w. The resultants were 6.14 ± 0.05, 45.67 ± 1.75 cp, 14.77 ± 1.19 min, 26.83 ± 2.15 MPa, 38.20 ± 2.39%, and 9.51 ± 0.19 mg/g, respectively. The optimal formulation of nicotine-containing plaster gel had a semicrystalline structure as it was derived from plant mucilage. It was immediately obvious that the formulation might control the release of nicotine, indicating first-order kinetic release. The *J*_ss_ and *K*_p_ values were 0.30 ± 0.01 mg/cm^2^/h and 3.13 ± 0.11 × 10^−2^ cm/h, respectively, indicating a maximum nicotine permeation of 78.82 ± 13.57%. When stored in a refrigerator as compared to room temperature, the nicotine-loading plaster gel thus showed excellent physical stability.

## 1. Introduction

The majority of industrial units employ synthetic polymers as a mediator for the formulation of the final products. Many plant-based excipients are currently accessible in the pharmaceutical industry, including gum, mucilage, pectin, gelatin, chitosan, cellulose, sugar, starch, agar, alginates, hyaluronate, and carrageenan. Pharmaceutics uses these green excipients as mediators for colloid, maintaining, thickening, gelling, and drug attachment. These excipients, which are biopolymers with long or branching chains, can expand when exposed to water [1].

Plants that include carbohydrates, proteins, and trace quantities of organic acids produce mucilage, which is a morpho-physiological product. Mucilage, which can frequently be produced from plants with normal growth as physiological metabolites within the cells, is useful to utilize as a binder and additive in medicinal and nutraceutical applications. Gums and mucilage are naturally occurring compounds that decompose, which makes them valuable for use in drug delivery systems [2–4]. Several research studies on mucilages derived from plants, such as *Aloe vera*, *Trigonella foenum-graecum*, *Abelmoschus esculentus*, *Dioscorea alata*, *Pereskia aculeata*, and numerous others have been done on a commercial scale [5, 6]. *Grewia ferruginea*'s mucilage is produced by extracting water from the inner stem's bark, which is then precipitated with ethanol, dried, and ground into a powder. *G. ferruginea* mucilage's ability to absorb moisture indicates the type of moisture present, and as temperature increased, so did the mucilage's solubility and swelling. *G. ferruginea* mucilage has an almost neutral pH. *G. ferruginea* mucilage can be used in pharmaceutical formulations as an adjuvant [7]. The mucilage of *Cassia obtusifolia* seeds is separated and assessed as a new biodegradable film for pharmaceutical use. Its polysaccharide content is assessed both quantitatively and subjectively. The results of the study conclusively show that films made from the mucilage of *C. obtusifolia* seeds have acceptable mechanical, thermal, and degrading characteristics for use in drug delivery applications [8].

*Phoenix dactylifera*, sometimes known as date palm, is a flowering plant in the palm family, Arecaceae, that is mostly farmed for its fruit. In the Middle East and North Africa, date fruits are a staple diet, yet there is still disagreement on where they originated. Nowadays, tropical and subtropical places all over the world are where it is mostly grown. Furthermore, it is currently eaten as food in a variety of global locations [9]. Date palm is important for nutraceuticals, but it also contains antibacterial, antimicrobial, antioxidant, anticarcinogenic, anti-hyperlipidemic, anti-inflammatory, antihyperglycaemic, antimutagenic, anticancer, and hepatoprotective properties. These characteristics have been correlated with phenolic substances, carotenoids, phytosterols, and phytoestrogens that were extracted from date palm products [10]. The most valuable source of mucilage is the fruit of the date palm. Date palm fruit that has been dried is a rich source of mucilage, which includes several reducing agents such as sugars and carbohydrates up to 88.01%. Mucilage also contains trace amounts of glucose, cellulose, and pectin, among other polysaccharides. When it comes to binding characteristics, date palm mucilage outperforms other gums [1, 11]. The ability of mucilage to bind and retain water is due to its high concentration of hydroxyl groups. Mucilage from the dried fruit of the date palm is a special and useful material. Its many possible uses in the food, pharmaceutical, and other industries have attracted increasing interest in recent years.

Plaster gel is a multipurpose substance that may be used in a variety of sectors and has a wide range of properties. When plaster gel is placed on the skin, it changes from a liquid gel to a film. A polymer matrix, such as polyvinyl alcohol (PVA) or hydroxypropyl methylcellulose (HPMC), that dissolves in a solvent or aqueous solution, is the usual component of plaster gel. Plasticizers, cross-linking agents, and reinforcing fillers are examples of additional additives that can be added to affect the mechanical, rheological, or adhesive characteristics of the gel [12]. Plaster gel adheres effectively to a wide range of substrates, including skin, mucosal surfaces, and porous materials. Because of its adhesive quality, it may be used in medical applications such as scaffolds for tissue engineering, transdermal drug delivery, and wound dressings [13].

The objective of designing and optimizing the nicotine-loaded plaster gel is to develop an effective transdermal drug delivery system using natural excipients, specifically *Phoenix dactylifera* (date palm) mucilage, PVA, and glycerin. While mucilage, a complex polymeric polysaccharide, serves as a natural excipient, its brittleness in film form may reduce flexibility, necessitating the incorporation of additional pharmaceutical excipients. To achieve an optimized formulation, response surface methodology (RSM) with Box–Behnken design (BBD) was applied, enabling systematic evaluation of formulation variables. The optimization process considered key factors such as pH, viscosity, drying time, mechanical properties, and drug content to ensure the development of a plaster gel with desirable characteristics for nicotine delivery. By providing controlled and sustained release, this formulation aims to enhance nicotine replacement therapy, reduce withdrawal symptoms, and improve patient compliance. This study highlights the potential of plant-based excipients in developing innovative and sustainable transdermal drug delivery systems.

## 2. Materials and Methods

### 2.1. Materials

The dried fruits of date palms were bought from the Rangsit market in Pathum Thani, Thailand. PVA (*M*_*w*_*M* 195,000) and (−)-Nicotine (with purity ≥ 99% for gas chromatography) were obtained from Sigma-Aldrich in the United States. The glycerin was purchased at the P.C. Drug Center in Thailand. We bought high-quality analytical grade chemicals from Fluka (USA), Sigma-Aldrich (USA), and Merck (Germany).

### 2.2. Preparation of Date Palm Mucilage

The process for preparing the mucilage of date palm was taken from an earlier investigation [5, 14, 15]. Overall, 4 L of distilled water was used to soak 500 g of dried fruits of date palms after they had been seeded, cut, and allowed for 36 h to dissolve. The solution was filtered through muslin cloth to remove any impurities. Diethyl ether was used for further purification after the mucilage was precipitated with 90% v/v ethanol. The filtered solution was then supplied with date palm mucilage and dried in a hot air oven at 40°C. A fine powder was mechanically milled from the dried mucilage and then preserved for subsequent use in an airtight container. The percentage of the yield was calculated using the following formula.

(1) $$\begin{array}{c} \text{Percentage of yield}=\frac{\text{weight of purified mucilage}}{\text{weight of soaked mucilage}}\times 100. \end{array}$$

### 2.3. Method Development and Optimization of Date Palm Mucilage With PVA-Based Plaster Gel

Three independent and six dependent variables were included in the BBD optimization of the suggested approach. When compared to other factorial approaches, BBD minimizes the number of experiments while providing higher-order response surfaces [16]. The date palm mucilage (A), PVA (B), and glycerin (C) were taken into consideration as independent factors in this design, whereas the pH value (*Y*_1_), viscosity (*Y*_2_), drying time (*Y*_3_), ultimate tensile strength (UTS; *Y*_4_), elongation at break (EB; *Y*_5_), drug content (*Y*_6_Y) were considered dependent variables. A preliminary investigation was used to define the maximum and lower limitations of these key parameters. The range of independent parameters that we chose for this experiment was as follows: the amount of date palm mucilage varied from 2% w/w to 4% w/w, the amount of PVA was defined between 0% w/w and 3% w/w, and the amount of glycerin got from 10% w/w to 30% w/w depending on the dry polymer content (Table 1). The statistical analysis was assessed, and experimental designs were generated using Design Expert® software (Version 11, Stat-Ease Inc., Minneapolis, MN, USA). There were 17 trial runs conducted in all, and Table 2 shows the responses for each. PVA pellets were dissolved in hot water and allowed to cool to room temperature before being used to prepare plaster gel. Glycerin and mucilage were combined and mixed. Then, 1% w/w of nicotine was added until a clear plaster gel was reached.

### 2.4. Evaluation of Date Palm Mucilage With PVA-Based Plaster Gel

#### 2.4.1. Appearance, Homogeneity, pH, and Viscosity

The plaster gels were assessed for homogeneity, viscosity, pH, and appearance. Visual inspection for bubbles and visible particles was used to gauge the plaster gel's homogeneity. Gel homogeneity was tested by pressing the gel between the thumb and index finger [17]. The pH of the plaster gels was tested at 25°C with a nonaqueous probe and a calibrated Mettler-Toledo electrode (Mettler-Toledo GmbH, Zurich, Switzerland) to guarantee that the formulations did not cause skin irritation. A digital viscometer (Brookfield model DV-II+, Stoughton, MA, USA) was used to measure the viscosity of plaster gel at 25 ± 1°C and 50 rpm spindle rotation.

#### 2.4.2. Liquid Drying Time

The drying time of the plaster gel was evaluated to mimic how well the product would work in real-life applications. A glass plate was covered with 0.5 g of liquid plaster, and this was the test method used. The weight change was then carefully measured using a 4-digit analytical scale (MS603S/01, Mettler-Toledo, Switzerland). Each experiment in the sample was run three times.

#### 2.4.3. Drug Content

The drug concentration was measured by ultrasonic method, which involved extracting 10 g of nicotine-containing plaster gel in distilled water for 30 min. The solution was withdrawn and diluted with distilled water to the appropriate concentration before being detected using a UV–visible scanning spectrophotometer (UV-1800, Shimadzu Corporation, Japan) that was recorded at 260 nm. Each experiment in the sample was run three times.

### 2.5. Characterization of Date Palm Mucilage With PVA-Based Plaster Gel

The obtained plaster gel containing nicotine was put onto a Petri dish about 30 g and dried overnight at 50°C in a hot air oven to remove the solvent from the formulation. The following characteristics of the obtained film were identified.

#### 2.5.1. UTS and EB

The TA.XT Plus texture analyzer (Texture Technologies Corporation and Stable Micro Systems, Ltd., USA) was utilized to perform the tensile test on each 1 cm × 6 cm film sample. The test cross-head speed was 10 mm/min, and the applied tensile force as a loaded cell was 500 N. The measuring gauge had a length of 1 cm × 1 cm [18]. The UTS was calculated by comparing the force of the load cell (N) with the cross-sectional testing area (width × thickness of the film in mm^2^). The length of the film at the breaking point was compared to its originating length to calculate the percentage of EB in millimeters. Each experiment in the sample was run three times [19].

#### 2.5.2. Differential Scanning Calorimetry (DSC)

The DSC equipment (DSC3+, Mettler-Toledo, Switzerland) was utilized to monitor the temperature behavior of the film sample at a rate of 10°C/min between 25°C and 400°C. The film sample was transferred to an aluminum pan, which was then hermetically sealed. DSC curves were used to determine and report the film sample's thermal characteristics.

#### 2.5.3. X-Ray Diffraction (XRD)

An XRD equipment (Empyrean, PANalytical, Netherlands) was used to verify the film sample's crystallinity. The parameters were adjusted at a 40 kV operational generator voltage and a 45 mA X-ray source current with a stepped angle of 0.02° (2*θ*)/s in the angular range of 5°–90° (2*θ*).

### 2.6. In Vitro Release of Nicotine From Date Palm Mucilage With PVA-Based Plaster Gel

For this work, the donor compartment of a modified Franz-type diffusion cell (Hanson® 57-6M, Hanson Research Corporation, USA) with an effective diffusion area of 1.77 cm^2^ was filled with 1 g of nicotine-containing plaster gel. The Franz cells were equipped with a dialysis cellulose membrane (MWCO: 3500 Da; CelluSep® T4, Membrane Filtration Products, Inc., USA). Before usage, the membrane was immersed in a receptor medium at 37 ± 0.5°C for a whole day. The receptor medium was 12 mL of an isotonic phosphate buffer solution at pH 7.4 with a water jacket at 37 ± 0.5°C. It was constantly stirred at 600 rpm using a magnetic stirrer. One milliliter of the receptor media was taken out at 0.5, 1, 2, 4, 6, 8, 10, 12, and 24 h, and it was replaced with an equivalent volume of fresh, pH 7.4 isotonic phosphate buffer solution. The UV–visible scanning spectrophotometer (UV-1800, Shimadzu Corporation, Japan) was used to measure the quantity of nicotine detected at 260 nm at room temperature. The experiment was performed three times on each sample. Equations (2)–(5) were used to calculate four models—zero-order, first-order, Higuchi's, and Korsmeyer–Peppas—to assess the kinetic release using DDSolver [20].

(2) $$\begin{array}{c} \text{Zero}-\text{order model }Q_{t}=Q_{0}+K_{0}t, \end{array}$$

(3) $$\begin{array}{c} \text{First}-\text{order model }\ln\;Q_{t}=\ln{\text{⁣}Q}_{0}‐{\text{⁣}K}_{1}t, \end{array}$$

(4) Higuchi's model Qt=KHt,

(5) $$\begin{array}{c} \text{Korsmeyer}-\text{Peppas model }\frac{Q_{t}}{Q_{0}}=K_{KP}t^{n}, \end{array}$$

where *Q*_0_ is the amount of initial drug and *Q*_*t*_ is the amount of drug released over time (*t*).

### 2.7. In Vitro Permeation of Nicotine From Date Palm Mucilage With PVA-Based Plaster Gel

For this work, the donor compartment of a modified Franz-type diffusion cell (Hanson® 57-6M, Hanson Research Corporation, USA) with an effective diffusion area of 1.77 cm^2^ was filled with 1 g of nicotine-containing plaster gel. A newborn-born pig skin that had naturally died (RSU-AEC 001–2024) was attached to the Franz cells. The receptor medium was 12 mL of an isotonic phosphate buffer solution at pH 7.4 with a water jacket at 37 ± 0.5°C. It was constantly stirred at 600 rpm using a magnetic stirrer. One milliliter of the receptor media was taken out at 0.5, 1, 2, 4, 6, 8, 10, 12, and 24 h, and it was replaced with an equivalent volume of fresh, pH 7.4 isotonic phosphate buffer solution. The UV–visible scanning spectrophotometer (UV-1800, Shimadzu Corporation, Japan) was used to measure the quantity of nicotine detected at 260 nm at room temperature.

The slope of the linear portion plot between the steady-state flow of the cumulative amount of nicotine permeated (mg/cm^2^) and time (*h*) was used to determine the permeation rate (*J*_ss_, mg/cm^2^/h). After that, the permeability coefficient (*K*_*p*_, cm/h) was obtained by dividing the flow by the initial loading of nicotine [21].

### 2.8. Stability of Date Palm Mucilage With PVA-Based Plaster Gel

The optimized plaster gel containing nicotine was stored at ambient temperature, refrigerated, and avoided light while its physical characteristics and drug content were monitored.

### 2.9. Statistical Analysis

Microsoft Excel was used to compute the means and standard deviations for every experiment. A one-way analysis of variance (ANOVA) was used to statistically analyze all the data, with a significance level of *p* < 0.05.

## 3. Results and Discussion

### 3.1. Optimization of Date Palm Mucilage With PVA-Based Plaster Gel

The physical properties of the crude date palm mucilage showed that, after purification, the dark brown color became light brown. This change might be the result of contaminants being removed. Furthermore, the physical characteristics changed from hard and spongy to glossy and crystalline. The investigation yielded a percentage of 50.26 ± 6.85% [15], which was less than the yield reported in the earlier publication [3, 5]. However, the film produced from date palm mucilage was brittle and had low elasticity; also, the film had an incomplete formation; hence, PVA and glycerin were added to solve these disadvantages. The BBD technique was used to further optimize the formulation to provide higher-order response surfaces with fewer investigations. Three independent components were considered: date palm mucilage (A), PVA (B), and glycerin (C), whereas the pH value (*Y*_1_), viscosity (*Y*_2_), drying time (*Y*_3_), UTS (*Y*_4_), EB (*Y*_5_), drug content (*Y*_6_) were considered dependent variables.

**Figure 1:**
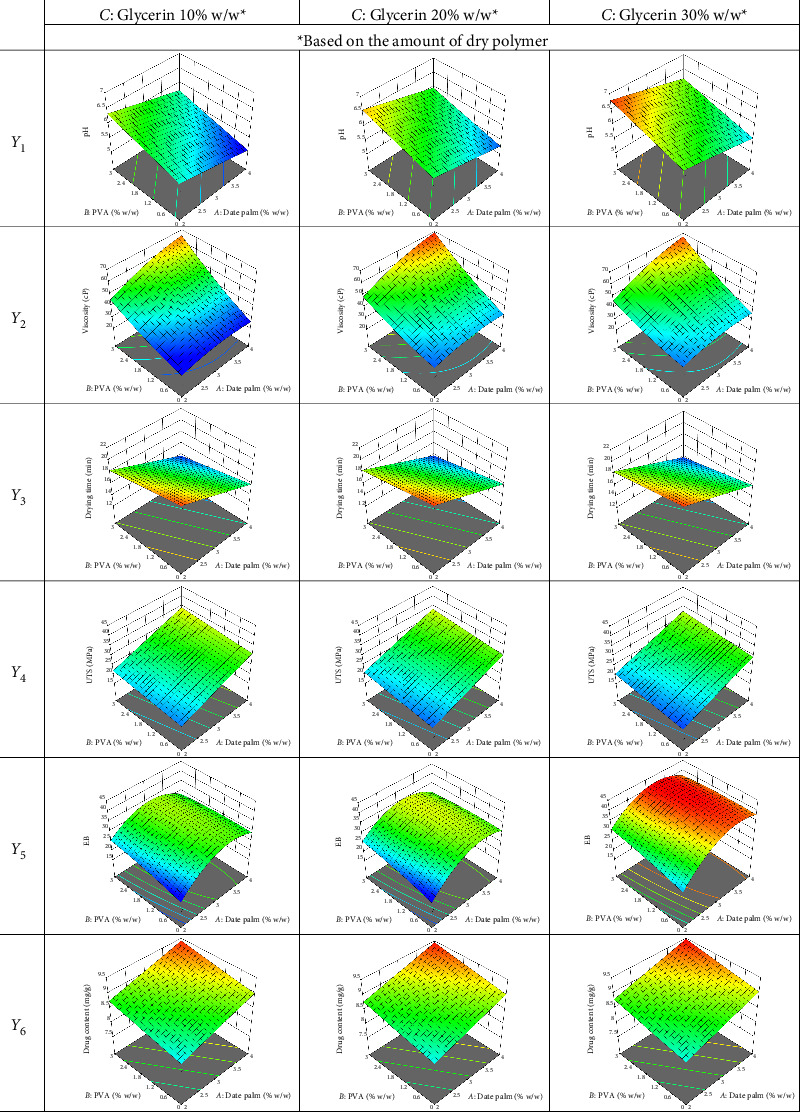
Response surfaces of dependent variables of plaster gel formulation.

It was discovered that all of the produced plaster gels were homogenous, satisfactory, and devoid of lumps or grit. Their color ranged from light to dark brown, depending on the amount of date palm mucilage. The optimization of ingredients for date palm mucilage with PVA-based plaster gel loading nicotine drug has been effectively achieved by the application of BBD. Table 2 summarizes the results of the ANOVA test, which revealed significant models for the chosen independent variables (*p* < 0.05). Six dependent variables (*Y*_1_–*Y*_6_) were shown to be significantly influenced by each of the independent variables, according to the ANOVA analysis. Three-dimensional response surface plots were generated based on these coded equations (Table 3) to show how the examined independent factors affected the dependent responses in Figure 1.

#### 3.1.1. pH Value (*Y*_1_)

Plaster gel's pH value demonstrated that the amount of date palm mucilage increased and that this caused the pH decrease, indicating that the solution was quite acidic. Plant mucilage is a complex polymeric substance that is mostly made up of carbohydrates and polysaccharides with highly branched structures. It also contains a variety of compounds in different portions, especially certain acids [7, 22]. Consequently, the resulting solution would seem rather acidic upon dissolving plant mucilage in water. The pH of plaster gel was significantly positively impacted by PVA and glycerin. This meant that as the concentration of glycerin and PVA increased, the formulation's pH value also increased. All formulations, however, had pH values between 5.0 and 7.0, which is the typical range for human skin [23]. They would consequently not irritate the skin, resulting in their safe to use.

#### 3.1.2. Viscosity (*Y*_2_)

The plaster gel's viscosity was dependent on the amounts of date palm mucilage, PVA, and glycerin. When date palm mucilage, PVA, and glycerin concentrations increased, the viscosity also increased. Furthermore, the viscosity was significantly positively impacted by the interactions between date palm mucilage and PVA as well as glycerin, but negatively impacted by the interactions between PVA and glycerin. It was also noteworthy to mention that polymers varying in concentration could form a gel and retain their viscosity over time. The viscosity of the nicotine-loaded plaster gel is significantly influenced by hydrogen bonding and polymer-polymer interactions among its components. Date palm mucilage, rich in hydroxyl (-OH) and carboxyl (-COOH) groups, forms strong hydrogen bonds with PVA, enhancing viscosity by creating a well-structured polymer network. Similarly, mucilage interacts with glycerin, contributing to viscosity retention through molecular entanglement and water-binding capacity [1, 24]. In contrast, PVA-glycerin interactions negatively impact viscosity, as glycerin, acting as a plasticizer, disrupts polymer-polymer bonding by intercalating between polymer chains and weakening intermolecular forces [25, 26]. This reduction in viscosity aligns with studies demonstrating that plasticizers interfere with hydrogen bonding in polymer matrices. Furthermore, variations in polymer concentration play a crucial role in gel formation and viscosity maintenance over time. Therefore, hydrogen bonding served as the primary mechanism governing viscosity changes, with mucilage-PVA and mucilage-glycerin interactions enhancing viscosity, while PVA-glycerin interactions diminish it. Moreover, because plaster gel formulations are applied to the thin layers of skin, consistency is one of the most significant features: the gel's viscosity plays a major influence in regulating drug absorption [27]. All plaster gel formulations were determined to have good skin adherence properties.

#### 3.1.3. Drying Time (*Y*_3_)

Drying time is the amount of time it takes for solvent components to evaporate and the formulation changes from a liquid gel to a film on the skin. The film formation time should thus be short and appropriate. Furthermore, drying time is affected by regional factors and weather conditions [13, 28]. The regional factors likely refer to environmental conditions such as temperature, humidity, and airflow, which influence the drying time of plaster gels. High temperatures and low humidity promote faster drying, while cooler temperatures and high humidity slow it down. Airflow also plays a role, with better ventilation accelerating the process [28]. The drying time was significantly impacted negatively by date palm mucilage and PVA, but positively by glycerin. This occurred when highly distilled water solvent was utilized in formulations with low polymer concentrations, resulting in slower evaporation. Moreover, most of the evidence indicated that glycerin hindered the evaporation of the solvent, which increased the amount of weight in the formulation and prolonged the drying time. This result corresponds to Raoult's law, which states that the vapor pressure above a solution will be lower than that of a pure solvent if any nonelectrolyte compounds are dissolved in it [29].

#### 3.1.4. UTS (*Y*_4_) and EB (*Y*_5_)

This experiment reported two different tensile properties: EB and UTS. The type and chemical composition of the components that produce films have an impact on their mechanical characteristics generally. The mechanical characteristics of films offer important information about their workability and application [30]. Although mucilage is a natural excipient composed of polymeric polysaccharides, it becomes brittle when produced into a film, potentially reducing its flexibility. Thus, the addition of PVA and glycerin would improve its disadvantage. In this research, date palm mucilage and PVA had a considerable positive impact on the UTS and EB, but glycerin had a negative influence. We hypothesize that an increase in network bonding between polymer-polymer was the cause of the stronger constructed film from the plaster gel formulation as the amount of polymer increased [31]. This suggested that the PVA addition was the cause of the films' strengthening effect. It is noteworthy that films constructed entirely of PVA exhibit a higher UTS than films that are compounded of other materials [24]. Increasing the amount of glycerin added to the formulation had varying effects on the mechanical properties of UTS and EB. The addition of glycerin improved the polymer chain mobility, which was expected to cause UTS decrease and EB increase. Plasticizing molecules, such as glycerin can reduce secondary indicates in polymeric chains by occupying the narrow intermolecular spaces between chains due to their low molecular weight [26, 32]. Furthermore, when the films were mechanically fractionated, an increase in glycerin concentration increased the enhancement of deformation. This phenomenon might likely be attributed to glycerin's capacity to decrease connections between polymeric chains, hence reducing resistance and enhancing film flexibility [25]. Additionally, the interactions between date palm mucilage and glycerin as well as PVA and glycerin had a significant desirable influence on the EB, but the interactions between date palm mucilage and PVA had a negative impact. Overall, the addition of PVA significantly enhanced the UTS and EB properties of the nicotine-containing plaster gel films by strengthening the network structure of the brittle mucilage-based films. These findings aligned with prior research, which highlighted PVA's superior mechanical reinforcement capabilities. However, increasing glycerin content reduced UTS while promoting EB, confirming glycerin's plasticizing effect. A previous study reported that PVA forms a robust matrix, enhancing UTS [24], while another highlighted the role of glycerin in reducing bonding resistance and increasing film flexibility [25]. These observations validate the present study's findings and underscore the compatibility of date palm mucilage with PVA and glycerin for producing mechanically optimized transdermal films.

#### 3.1.5. Drug Content (*Y*_6_)

It was found that the drug concentration in the plaster gel formulation was positively caused by the three independent ingredients of date palm mucilage, PVA, and glycerin. It might have been concluded that as the amount of polymer in the formula increased so did the viscosity and capacity for holding the drug. These values might lower than the ideal amount of drug loading. A lower entrapment value may be the consequence of some drugs being firmly entrapped in the formulation and being unable to be entirely removed from the formulation [33]. Nonetheless, research demonstrated that the drug molecules remained present in the formulations throughout the production process without any loss.

In addition, Table 3 shows that the average prediction error is compared to the range of expected values at the design points, and the signal-to-noise ratio is assessed (a ratio greater than 4 is judged acceptable) [34]. There was no need for further testing because the ratio was more than 4, indicating that the model could project outcomes inside its targeted region.

**Figure 2:**
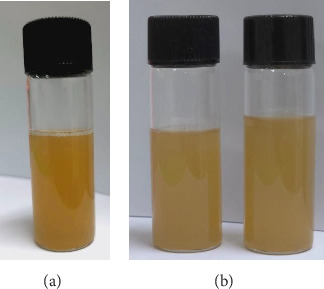
Appearance of nicotine-loading plaster gel: (a) fresh preparation and (b) stability test. Stability test 4 weeks: (left) ambient temperature and (right) refrigerator.

The effects of components and interactions on their final results were impacted when the quantities of date palm mucilage, PVA, and glycerin increased. This was evident in the summary of all dependent variables. The experiment was designed using a statistical strategy to quickly optimize system performance using known parameter values. A screening experimental design procedure comprising all known parameters suspected of influencing the system's performance is the first step in the process. Reducing the number of research elements to a manageable few is an important objective of the experiment when there are too many of them. Another planned experiment or test plan targeted at enhancing the system's performance usually follows this [35–37]. Because we were planning on using PVA to help produce a completed film of mucilage, it should be used in small amounts to get the ideal formula. Overall, based on Figure 1 and Table 2, the highest viscosity and pH values were preferred because they allowed for skin contact while the plaster gel was still gel-like and allowed for the application of the gel on the skin without irritating [38]. Because it would quickly form a film on the skin, the time required for drying should be as short as possible. There should be adequate flexibility, or low TS and high EB values after the plaster gel has created a thin film on the skin. Lastly, there should be the highest amount of drug finding possible in the formulation. Consequently, the criteria for optimizing nicotine-loading plaster gel are summarized in Table 1.  Table 4 shows that this study was effective in reducing the number of trials necessary for producing the plaster gel formulation including date palm mucilage, PVA, and glycerin at a weight ratio of 3.5%:1.8%:30% w/w, respectively. The maximum desirability value for a weight ratio was 1.000. The appropriate weight ratio was established again to confirm the accuracy of Design-Expert's predictions, as shown in Table 4. The percentage of errors varied between 1.17% and 10.41%. These results meant that the predictions made by Design-Expert had a low proportion of error and were correct. For the next pharmaceutical application study, the ideal weight ratio of date palm mucilage, PVA, and glycerin—3.5%:1.8%:30% w/w—was thus used. Light brown was the color of the produced optimum plaster gel containing nicotine (Figure 2(a)).

### 3.2. Characterization of Fabricated Film of Date Palm Mucilage With PVA-Based Plaster Gel

#### 3.2.1. Liquid Drying Time

**Figure 3:**
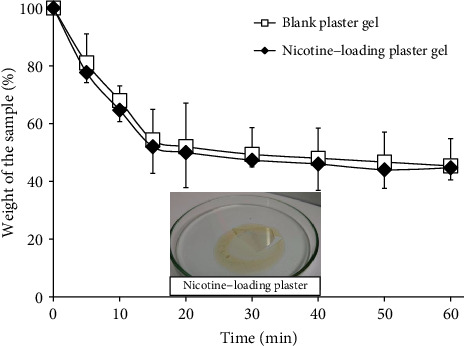
Drying time profiles of blank plaster gel and nicotine-loading plaster gel.

A profile of the plaster gel drying times is presented in Figure 3. There was no significant difference seen in the drying times of nicotine-loading plaster gel and blank plaster gel. All formulations had fast evaporation rates within the first 10 minutes, which were estimated to be responsible for 30 percent of the weight loss. Subsequently, the solvent would progressively evaporate at its evaporation rate. It took approximately 15 min to produce a complete film. The dry film formed at 45.33 ± 4.16% and 44.67 ± 9.45% of the total weight of blank and nicotine-loaded plaster gel, respectively.

#### 3.2.2. DSC

**Figure 4:**
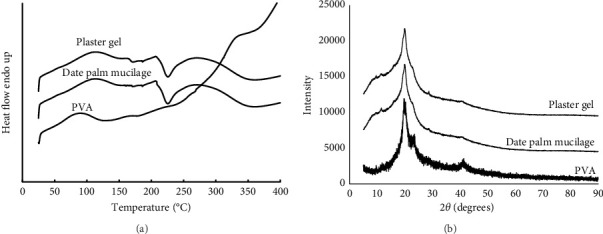
(a) DSC thermogram and (b) XRD pattern of nicotine-loading plaster gel.

A thermal method known as DSC is used to investigate the glass transition temperature (*T*_*g*_), melting temperature (*T*_*m*_), and crystallization temperature (*T*_*c*_), in addition to the enthalpy that is connected with each step. For pure nicotine, the DSC curve shows an endothermic thermal event at 242.68°C. This temperature corresponds to the melting point of nicotine, which is reported in the previous work [39]. The characteristic Tm of nicotine, on the other hand, did not appear in the DSC curve that we presented in Figure 4(a). Although this curve did not show nicotine characteristics, it was discovered by extracting and analyzing it with HPLC. This was due to the simple reason that the formulation contained a significantly lower quantity of nicotine than other constituents. There was a possibility that the DSC curve of the nicotine-loading plaster gel might only be shown for polymer blends in the formulation (Figure 4(a)). Two regions of thermal characteristics were observed, and those regions were *T*_*g*_ and *T*_*c*_. The first peaks that were seen occurred at 108.17°C, and the energy of the reaction was 113.55 J/g, which corresponds to the *T*_*g*_. The thermogram in the present case began at 45.01°C and concluded at 161.66°C. This indicates that water evaporation occurred, and the heating produced a large endothermic peak, which is characteristic of organic compound materials with lower molecular weights or short chains that include hydrophilic groups seen in plant mucilage [40]. The semicrystalline structure of the mucilage was demonstrated by the second peak, which was found to occur at 225.67°C and where the energy of the reaction was 46.01 J/g. This peak corresponds to the *T*_*c*_ of the formulation.

#### 3.2.3. XRD

The amorphous, semicrystalline, or crystalline structure of the film of the plaster gel was investigated using XRD. The XRD pattern of nicotine-loading plaster gel is seen in Figure 4(b). The XRD pattern indicated the high-intensity peak of the semibroad peak found at 20.08° which supported the semicrystalline structure of the plant mucilage. This XRD observation was in agreement with the DSC result that indicated a semicrystalline structure. Plant mucilage has several hydroxyl groups that may be advantageous for the formation of hydrogen-bonded interactions between and within molecules, resulting in variability in the mucilage's crystallinity [40, 41]. Because of its semicrystalline structure, mucilage may be used as a thickening, strengthening, and possibly reinforcing agent [42].

### 3.3. In Vitro Release

**Figure 5:**
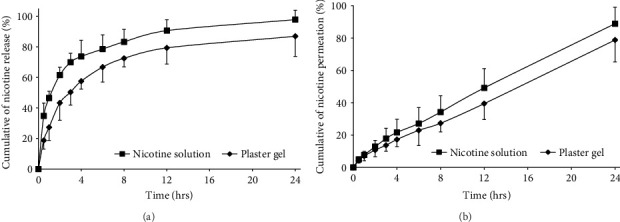
(a) In vitro release and (b) in vitro permeation of nicotine from plaster gel.

The initial concentration of nicotine in the plaster gel was 9.51 ± 0.19 mg/g. Consequently, a nicotine solution of 10 mg/g was prepared in distilled water to be equivalent to the nicotine content in the formulation and placed in the donor compartment. The profiles of nicotine release are shown in Figure 5(a). Pure nicotine was faster released into the medium from distilled water than from the plaster gel formulation. This resulted from the possibility that the gel might delay the release of nicotine. The highest nicotine release observed in a 24-h study was 97.80 ± 6.13% for distilled water and 86.89 ± 13.15% for plaster gel formulation. The delay in nicotine release from the plaster gel formulation was attributed to the structural and compositional characteristics of the gel, specifically the interactions between date palm mucilage, PVA, and glycerin. These components formed a cohesive polymer matrix that regulated drug diffusion through several mechanisms. Firstly, date palm mucilage, rich in polysaccharides and hydroxyl groups, formed a semicrystalline network with a high capacity for hydrogen bonding. This network created a diffusion barrier, which hindered the rapid release of nicotine molecules. The semicrystalline structure, as evidenced by XRD pattern, supported a slower release by limiting free molecular movement [43]. Secondly, PVA contributed to the formation of a robust polymer matrix, further reinforcing the diffusion barrier. Its hydrogen-bonding interactions with mucilage increased the gel's structural integrity and created a tightly knit network that restricted nicotine diffusion. The role of PVA as a diffusion-controlling agent had been demonstrated in previous studies, where it provided sustained drug release due to its crystalline domains [44]. Lastly, glycerin acted as a plasticizer, enhancing film flexibility but simultaneously contributing to the gel's water-retention properties. This slowed down solvent evaporation and helped maintain the gel matrix, which further moderated the diffusion of nicotine. Similar findings were reported in formulations where glycerin prolonged the release of active compounds by increasing gel moisture content and stability [45]. The combined effects of these interactions—mucilage's semicrystalline nature, PVA's network reinforcement, and glycerin's water-retention properties—created a diffusion-controlled environment. This mechanistic understanding aligned with studies demonstrating how polymeric gel matrices modulated drug release profiles through diffusion and polymer relaxation processes. Thus, the gel's ability to delay nicotine release was attributed to these complex interactions, which maintained a controlled and sustained release environment.

The DDSolver application is utilized to calculate the kinetic releases (Table 5). It was discovered that the first-order model with the highest *R*^2^ provided the appropriate kinetic release of nicotine from both distilled water and plaster gel formulation. The fact that the release was proportional to the amount of nicotine remaining in the formulation and time-dependent was also readily evident. The release rate of nicotine from distilled water was approximately twice that of the plaster gel formulation. The Korsmeyer–Peppas model has the benefit of allowing for mechanistic problems such as modeling the drug release process from formulation using the transport exponent, *n*. When there are no limits, *n* must equal or be less than 0.5 to have a Fickian diffusion process; on the other hand, values of *n* between 0.5 and 1 show non-Fickian diffusion and suggest the presence of a boundary region influencing passive drug diffusion [46]. The Korsmeyer–Peppas model predicted that the diffusion exponent *n*-value for nicotine release from the plaster gel formulation was 0.323, which might be explained by Fickian diffusion. In summary, it was found that the release of nicotine from the plaster gel formulation was probably caused by a diffusion-controlled process.

### 3.4. In Vitro Permeation

An in vitro permeation study was performed to assess the nicotine's effectiveness to enter the dermis and *epidermis* of dead pig skin. The profiles of nicotine permeation are shown in Figure 5(b). The highest nicotine permeation observed in a 24-h study was 88.92 ± 10.13% for distilled water and 78.82 ± 13.57% for plaster gel formulation. In vitro procedures using animal skin are frequently used to examine the penetration of potentially topical drugs. The permeability features of the stratum corneum, which may be considered as the rate-limiting step for skin absorption, remain unchanged after removal from the body, allowing for direct comparison [47]. Nicotine might permeate the *epidermis* to a limited extent, despite having a high release profile. The calculated parameters for the in vitro nicotine permeation from distilled water, *J*_ss_, and *K*_*p*_ were 0.35 ± 0.02 mg/cm^2^/h and 3.57 ± 0.17 × 10^−2^ cm/h, respectively, whereas the values from plaster gel formulation were 0.30 ± 0.01 mg/cm^2^/h and 3.13 ± 0.11 × 10^−2^ cm/h, respectively.

In our study, the plaster gel formulation exhibited a sustained nicotine release, with approximately 86.89% of nicotine released over a 24-h period following a first-order kinetic model. This controlled release profile contrasts with the faster release observed from a pure nicotine solution, indicating that the natural polymeric network—formed by date palm mucilage, PVA, and glycerin—effectively retards drug diffusion. Such a mechanism is largely attributed to the semicrystalline structure of the gel, where extensive hydrogen bonding within the mucilage-PVA matrix creates a diffusion barrier. Furthermore, the in vitro permeation studies revealed a maximum nicotine permeation of 78.8% over 24 h, with *J*_ss_ and *K*_*p*_ values of 0.30 mg/cm^2^/h and 3.13 × 10^−2^ cm/h, respectively. When comparing these findings with conventional transdermal nicotine patches—typically formulated using synthetic polymers—it is evident that our formulation achieves a slower, more controlled permeation profile. Literature reports often highlight that synthetic polymer-based systems tend to exhibit higher initial fluxes and faster overall permeation rates [39, 48]. The novelty of our approach lies in the use of a natural, plant-derived mucilage from *P. dactylifera* (date palm), which not only contributes to the eco-friendly and sustainable nature of the formulation but also imparts unique physicochemical properties. The natural mucilage, with its high concentration of hydroxyl groups, enhances the formation of a robust, semicrystalline network. This network is key in moderating both the release and permeation of nicotine, thereby potentially reducing the likelihood of peak-related side effects often associated with more rapid drug delivery. By situating our findings within the broader context of nicotine delivery research, it becomes clear that the incorporation of date palm mucilage provides a promising alternative to traditional synthetic systems. The controlled release and moderate permeation parameters observed in our study may translate into improved patient compliance and a reduced risk of adverse effects, offering a novel pathway for NRT.

### 3.5. Stability

**Figure 6:**
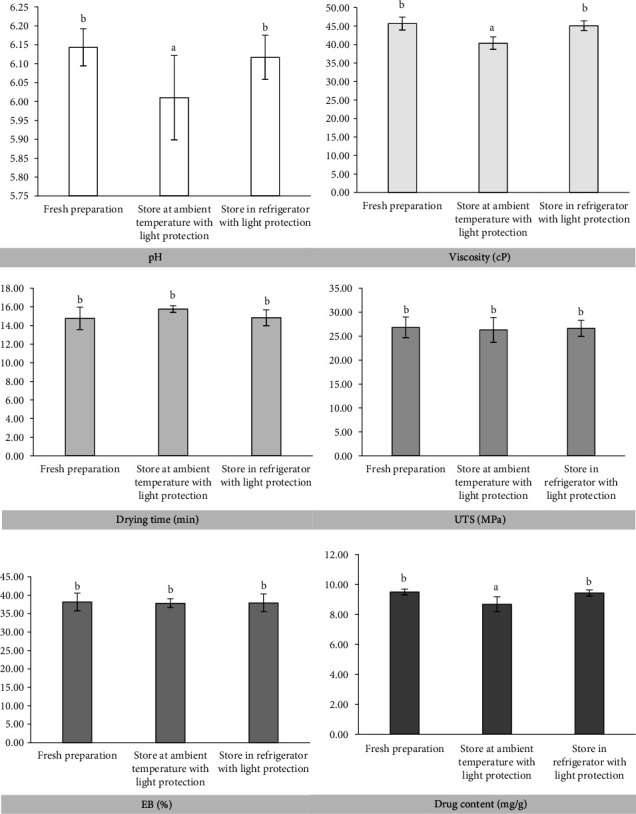
Properties of the produced optimum plaster gel containing nicotine after the stability test. (*a* = *p* < 0.05 and *b* = *p* > 0.05).

The plaster gel formulation was found to be stable after 4 weeks of storage, as evidenced by the absence of color changes in tests performed both at ambient temperature and in a refrigerator with light protection (Figure 2(b)).  Figure 6 shows the product's characteristics, including pH, viscosity, and drying time, indicating modest variations; however, these might be better controlled by storage in a refrigerator instead of at the ambient temperature. Surprisingly, there was no significant variation in mechanical properties between UTS and EB following storage under both conditions compared to fresh preparation. Although storage would have nearly identical effects, nicotine became available in the formulation after stability tests revealed that keeping it in the refrigerator maintained more of the drug than keeping it at ambient temperature. The amount of nicotine that remained in the plaster gel formulation decreased due to its volatility and potential for fast degradation when exposed to weather conditions. These results therefore showed that the nicotine-loading plaster gel showed high physical stability with little alterations noted; we highly recommended keeping it in a refrigerator rather than at ambient temperature.

Although our study included stability tests under both ambient and refrigerated conditions over a 4-week period, a more in-depth evaluation of the long-term stability and shelf life of the nicotine-loaded plaster gel is essential for its practical application. Extended stability studies should consider the following aspects:

1. Nicotine degradation: Over longer storage periods, nicotine is prone to degradation due to its inherent volatility and susceptibility to oxidative processes. To ensure therapeutic efficacy, it is crucial to monitor the degradation rate of nicotine using advanced analytical techniques such as HPLC. Future studies should include accelerated stability testing under controlled conditions (e.g., 40°C/75% RH) over six to 12 months, which will help predict the shelf life and establish appropriate storage conditions.
2. Gel consistency: The physical consistency of the gel, including its viscosity and drying time, is expected to remain stable to guarantee consistent drug release performance. Extended studies should assess whether any phase separation or polymer degradation occurs over time, which could alter the gel network. Regular evaluations of viscosity and film formation properties will provide insight into potential changes in gel structure that might affect both application and drug release.
3. Mechanical properties: Mechanical integrity, characterized by parameters such as UTS and EB, is critical for ensuring the durability and user comfort of the plaster gel. Prolonged storage might lead to a decline in these mechanical properties due to ongoing molecular rearrangements or loss of plasticizer efficacy. Therefore, long-term assessments should monitor these properties periodically to confirm that the film maintains adequate flexibility and strength throughout its intended shelf life.

In summary, while initial stability tests indicate that the formulation remains robust over a short period, comprehensive long-term stability studies are necessary. These studies should focus on quantifying nicotine degradation, maintaining gel consistency, and preserving mechanical properties to reliably predict the formulation's shelf life. Such data will be instrumental in guiding storage recommendations and ensuring the consistent performance of the nicotine-loaded plaster gel during its entire lifecycle.

## 4. Conclusions

The most important objective of this research was to optimize the various components utilized in the nicotine-containing plaster gel of a plant mucilage obtained from the fresh fruit of *P. dactylifera* (date palm), with an emphasis on assessing its practical properties. The physicochemical features of the resultant (pH value, viscosity, drying time, UTS, EB, and drug content) were found to be influenced by a variety of factors, including date palm mucilage, PVA, and glycerin. The results showed that the date palm mucilage, PVA, and glycerin at a weight ratio of 3.5%:1.8%:30% w/w were beneficial in lowering the number of trials required to produce the nicotine-containing plaster gel formulation. The optimal nicotine-containing plaster gel formulation indicated about 15 min to make a complete film that formed at 44.67 ± 9.45% of the total weight of the dried film. Because it was made from plant mucilage, the optimal formulation of nicotine-containing plaster gel had a semicrystalline structure. It was also easily apparent that the formulation could regulate the release of nicotine by making it proportionate to the total amount of nicotine remaining in the formulation and time-dependent. The highest nicotine permeation found in a 24-h investigation was 78.82 ± 13.57%. In vitro nicotine permeation yielded *J*_ss_ and *K*_*p*_ values of 0.30 ± 0.01 mg/cm^2^/h and 3.13 ± 0.11 × 10^−2^ cm/h, respectively. The nicotine-loading plaster gel consequently demonstrated good physical stability with few changes seen, according to stability testing; we strongly advised storing it in a refrigerator instead of room temperature.

## Figures and Tables

**Table 1:** Experimental design.

	Level		
	Low (−1)	Medium (0)	High (+1)
*Factors (independent variables)*			
*A*: Date palm mucilage (% w/w)	2	3	4
*B*: PVA (% w/w)	0	1.5	3
*C*: Glycerin^∗^ (% w/w)	10	20	30

*Responses (dependent variables)*			
*Y*_1_: pH	Maximize		
*Y*_2_: Viscosity (cP)	Maximize		
*Y*_3_: Drying time (min)	Minimize		
*Y*_4_: UTS (MPa)	Minimize		
*Y*_5_: EB (%)	Maximize		
*Y*_6_: Drug content (mg/g)	Maximize		

^∗^Based on the amount of dry polymer.

**Table 2:** Three-level Box–Behnken design experiments are conducted with actual values and observed responses.

Runs	*A* (% w/w)	*B* (% w/w)	*C* ^∗^ (% w/w)	*Y*_1_	*Y*_2_ (cP)	*Y*_3_ (min)	*Y*_4_ (MPa)	*Y*_5_ (%)	*Y*_6_ (mg/g)
F1	3	1.5	20	5.93 ± 0.15	48.63 ± 4.04	17.60 ± 0.62	26.70 ± 2.82	36.63 ± 1.02	8.86 ± 0.34
F2	3	1.5	20	5.90 ± 0.70	43.43 ± 3.76	17.20 ± 0.36	27.67 ± 1.36	35.33 ± 1.34	9.03 ± 0.20
F3	4	3	20	6.33 ± 0.47	69.60 ± 3.04	13.53 ± 0.70	40.50 ± 1.47	27.70 ± 1.35	9.45 ± 0.53
F4	2	1.5	30	6.67 ± 0.25	37.53 ± 3.79	19.47 ± 1.01	20.37 ± 1.12	29.17 ± 1.90	8.67 ± 0.28
F5	3	1.5	20	5.60 ± 0.26	49.33 ± 5.03	16.90 ± 0.66	26.07 ± 0.25	36.40 ± 2.03	9.10 ± 0.05
F6	4	0	20	5.13 ± 0.25	35.07 ± 3.00	14.43 ± 1.07	29.03 ± 2.10	32.10 ± 3.13	9.00 ± 0.58
F7	4	1.5	10	5.63 ± 0.25	42.97 ± 1.70	16.13 ± 0.70	29.37 ± 1.63	28.67 ± 1.35	9.14 ± 0.64
F8	3	1.5	20	5.53 ± 0.40	45.27 ± 4.32	16.37 ± 0.55	25.23 ± 3.01	35.27 ± 1.21	8.98 ± 0.31
F9	2	3	20	6.87 ± 0.25	46.93 ± 2.47	18.50 ± 0.98	21.97 ± 2.73	24.73 ± 1.78	8.70 ± 0.38
F10	2	1.5	10	6.20 ± 0.20	33.63 ± 3.37	17.87 ± 0.61	17.40 ± 0.95	20.07 ± 1.65	8.68 ± 0.24
F11	4	1.5	30	6.10 ± 0.17	47.93 ± 3.86	14.43 ± 0.71	25.73 ± 1.50	41.33 ± 3.83	9.12 ± 0.18
F12	3	3	10	5.50 ± 0.20	58.50 ± 2.69	16.47 ± 0.80	26.70 ± 1.83	37.67 ± 1.89	8.89 ± 0.18
F13	2	0	20	5.87 ± 0.15	29.97 ± 2.99	20.93 ± 0.80	15.47 ± 1.36	21.93 ± 2.90	7.94 ± 0.38
F14	3	3	30	6.43 ± 0.40	60.60 ± 3.52	16.33 ± 1.05	22.13 ± 2.05	41.13 ± 4.52	9.19 ± 0.35
F15	3	0	10	5.73 ± 0.15	26.43 ± 3.65	18.87 ± 0.74	27.43 ± 0.99	31.37 ± 3.46	8.81 ± 0.26
F16	3	0	30	5.70 ± 0.30	35.93 ± 3.04	19.40 ± 0.70	26.40 ± 0.90	34.47 ± 1.65	8.85 ± 0.18
F17	3	1.5	20	5.60 ± 0.20	44.60 ± 2.76	16.93 ± 1.10	29.10 ± 1.67	28.40 ± 2.14	8.78 ± 0.33

^∗^Based on the amount of dry polymer.

**Table 3:** Response mathematical models and actual equations.

Responses	Mathematical models	Actual equations	Adeq precision^∗^
*Y*_1_: pH	Linear	*Y*_1_ = 6.03−0.30(*A*) + 0.23(*B*) + 0.02(*C*)	8.4250
*Y*_2_: Viscosity (cP)	Quadratic	*Y*_2_ = −14.36 + 18.15(*A*) + 0.04(*B*) + 1.51(*C*) + 2.92(*A* ∗ *B*) + 0.03(*A* ∗ *C*) − 0.12(*B* ∗ *C*) − 2.86(*A*^2^) + 0.89(*B*^2^) − 0.03(*C*^2^)	23.0321
*Y*_3_: Drying time (min)	Linear	*Y*_3_ = 25.00 − 2.28(*A*) − 0.73(*B*) + 0.03(*C*)	16.1191
*Y*_4_: UTS (MPa)	Linear	*Y*_4_ = 7.13 + 6.18(*A*) + 1.08(*B*) − 0.08(*C*)	9.1048
*Y*_5_: EB (%)	Quadratic	*Y*_5_ = −41.14 + 46.67(*A*) + 5.39(*B*) − 0.91(*C*) − 1.20(*A* ∗ *B*) + 0.09(*A* ∗ *C*) + 0.01(*B* ∗ *C*) − 7.07(*A*^2^) − 0.32(*B*^2^) + 0.02(*C*^2^)	7.2771
*Y*_6_: Drug content (mg/g)	Linear	*Y*_6_ = 7.60 + 0.34(*A*) + 0.14(*B*) + 0.01(*C*)	14.3458

^∗^The Adeq precision value measure assesses the signal-to-noise ratio. A ratio greater than 4 is preferred, indicating that this model can be utilized to explore the design space without the need for additional tests.

**Table 4:** Predicted value, experimental value, and percentage error of plaster gel.

*A* (% w/w)	*B* (% w/w)	*C* ^∗^ (% w/w)	*Y*_1_	*Y*_2_ (cP)	*Y*_3_ (min)	*Y*_4_ (MPa)	*Y*_5_ (%)	*Y*_6_ (mg/g)
3.5	1.8	30	Predicted value from Design-Expert® program					
			6.07	50.97	15.86	28.26	41.33	9.13
			Experimental value					
			6.14 ± 0.05	45.67 ± 1.75	14.77 ± 1.19	26.83 ± 2.15	38.20 ± 2.39	9.51 ± 0.19
			Percentage error^∗∗^					
			1.17	10.41	6.87	5.06	7.58	4.14

^∗^Based on the amount of dry polymer.

^∗∗^Percentage error = [(Experimental value − predicted value/experimental value) × 100].

**Table 5:** Kinetic models of nicotine release from plaster gel using add-in DDSolver program.

	*R* ^2^			Release rate ∗ *K*_1_ (h^−1^)	*n*
	Zero-order model	First-order model	Higuchi's model		Korsmeyer–Peppas model
Nicotine solution	0.7255	0.9843	0.9011	0.422	—
Plaster gel	0.7989	0.9863	0.9461	0.205	0.323

^∗^Calculated from best kinetic (first-order model).

## Data Availability

The data that support the findings of this study are available from the corresponding author upon reasonable request.
